# Influence and Mechanism of Curing Methods on Mechanical Properties of Manufactured Sand UHPC

**DOI:** 10.3390/ma15186183

**Published:** 2022-09-06

**Authors:** Chengfang Yuan, Shiwen Xu, Ali Raza, Chao Wang, Di Wang

**Affiliations:** 1 College of Water Resources and Civil Engineering, Zhengzhou University, Zhengzhou 450001, China; 2 Yellow River Laboratory, Zhengzhou University, Zhengzhou 450001, China

**Keywords:** manufactured sand, UHPC, curing methods, mechanical properties, bond strength

## Abstract

The mechanical properties of ultra-high performance concrete (UHPC) made of manufactured sand (MS) under four curing methods (steam, standard, sprinkler and saturated Ca(OH)_2_) were investigated via compressive, flexural and uniaxial tensile tests, and the bond strength of steel fiber and manufactured sand UHPC (MSUHPC) matrix. Based on the analysis of the microstructure, the influence mechanism of curing methods on the mechanical properties of materials was explored. The results showed that the early compressive strength of MSUHPC under steam curing (SM) is much higher than that of the other three curing methods, but the difference gradually decreases with the increase of age. The compressive strength of MSUHPC under SM is higher than that of river sand UHPC (RSUHPC). The bending strength and compressive strength of MSUHPC under different curing methods are similar, and the bending strength of 28 days steam cured samples is the highest. The uniaxial tensile properties of MSUHPC did not show significant difference under standard curing (SD), sprinkler curing (SP) and saturated Ca(OH)_2_ curing (CH), and the uniaxial tensile properties of MSUHPC under SM are slightly better than RSUHPC. The ultimate bond strength and fiber pullout energy of steel fiber and MSUHPC increase with the development of age. The bond strength and fiber pullout work of SM is higher than those of the other three curing methods, but there are lower increases in the later stage than that of the other three curing methods.

## 1. Introduction

Since the second half of the 20th century, with the rapid development of engineering structures and economic growth, people also have new requirements for the performance of engineering materials, and have begun to explore and research high performance and even ultra-high performance engineering materials. In 1994, the concept of ultra-high performance concrete (UHPC) was proposed [1]; then the research on reactive powder concrete was published and had a warm response in the field of civil engineering, which marked the entry of concrete into the era of ultra-high performance [2].

UHPC is a high density cement-based composite material with high strength and high ductility, which has been applied in maintenance and reinforcement, bridge engineering and other fields [3,4,5,6,7,8,9]. It is well known that the manufacturing of UHPC requires a large quantity of natural river sand (RS) as fine aggregates. However, natural RS is increasingly scarce due to the exhaustive exploitation. Therefore, it is urgent to find other raw materials to replace natural sand in producing UHPC. Manufactured sand (MS) is a kind of artificial fine aggregate from natural stone based on a series of breaking and grinding techniques, which could be processed in larger amount and lower cost [10,11,12,13,14,15,16]. Studies have shown that MS can replace natural sand to prepare UHPC and be used in practical engineering [17,18,19,20,21,22,23]. Yoo et al. [24] studied the mechanical properties of UHPC under various curing methods and found that thermal curing can promote hydration reaction and improve the compressive strength of UHPC. Zdeb [25] found through microstructural studies that with the increase of temperature, C-S-H will produce a new crystalline hydrate, which makes the structure of concrete denser, the temperature increases by 10 °C, and the compressive strength of concrete increases by 16 MPa. Yazıcı et al. [26] studied the influence of auxiliary cementitious materials and curing conditions on the compressive strength of UHPC, and found the optimal dosage of different auxiliary cementitious materials and optimal curing conditions. Wu et al. [27] investigated the mechanical properties of UHPC under three curing methods. The results showed that when the curing time is same, hot water curing and SM resulted in higher compressive strength and bending strength of UHPC than that of standard curing. However, Shi et al. [28] conducted studies on hydration products, microscopic morphology, pore structure and interfacial transition zone (ITZ), and the results showed that SM can adversely affect the long-term performance of concrete by changing the internal temperature and humidity field. Liu et al. [29] found through the study of pore structure that although SM can adversely affect the long-term performance of concrete, proper subsequent wet curing can effectively reduce the adverse effects of SM on concrete. At the same time, Ballim Y. [30] measured the oxygen permeability and water absorption at different depths in the concrete, and the result showed that wet curing had a protective and sustainable effect on the durability of concrete.

The curing method of concrete has an important influence on the formation of its strength and later development. The commonly used curing method for UHPC is high-temperature steam curing, which is relatively complex and more suitable for preparing prefabricated components in fixed places such as prefabricated yards [31]. For some components that need to be poured on site, it is generally difficult to achieve the conditions of SM due to site factors. Therefore, it is also very important to study the mechanical properties of UHPC under other normal temperature curing conditions.

Gao H et al. [32] studied the case of concrete CO_2_ erosion; by using a Ca(OH)_2_ solution, wetting concrete can improve its density and compressive strength, and this method can solve the problem of CO_2_ erosion of cement and concrete materials.

Existing research results have shown that different curing conditions have a great influence on the hydration reaction rate and physical and mechanical properties of UHPC [33,34]. The object of this study is to gain insights into the mechanical and microstructural properties of manufactured sand UHPC (MSUHPC) under different curing methods. The effects of four curing methods (steam curing (SM), standard curing (SD), sprinkler curing (SP) and saturated Ca(OH)_2_ curing (CH)) on mechanical and microstructural properties of concrete are investigated. It is greatly significant to promote the application of MS in UHPC preparation.

## 2. Materials and Methods

### 2.1. Material

**Figure 1:**
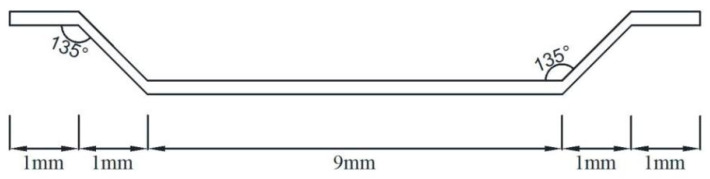
Shape diagram of steel fiber.

This study adopted ordinary Portland cement (P·O52.5) produced by Mengdian Cement Industry (Huixian, Henan, China). The physical properties of the cement are shown in Table 1. Fly ash produced by Hengnuo Filter Material (Gongyi, China) and silica fume (SiO_2_ content 97%) produced by Hengyuan Material (Xining, Qinghai, China) were used. The steel fiber was made of copper-plated steel fiber produced by Sida Steel Fiber Industry (Tengzhou, China). The shape diagram is shown in Figure 1, and the specifications and performance indicators are shown in Table 2. Two types of fine aggregate (MS and RS) were applied here. The test reports are shown in Table 3 and Table 4. The test equipment, methods and calculations are in accordance with the specification JGJ 52-2006 [35]. Polycarboxylic superplasticizer with water-reducing of 26% was produced by Chenqi Industy (Shanghai, China). Common tap water was used for mixing and maintenance.

### 2.2. Sample Preparation

**Figure 2:**
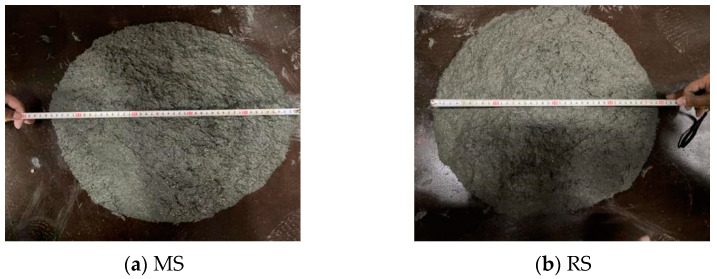
Concrete slump spread test.

The workability of concrete is characterized by the slump of the specification GB/T 50080-2016 [37]. The test process is shown in Figure 2, and the mix proportion and slump spread are listed in Table 5. UHPC mixture was mixed by SJD60 forced mixer. First, the inner wall of the mixer was wiped with wet cloth. During mixing, dry powders, including cement, silica fume, fly ash, superplasticizer and expansive agent were mixed for 1–2 min. Water was then added and mixed for approximately 5 min. MS or RS was then added and mixed for approximately 3 min. Steel fibers were added uniformly and mixed for 3 min after forming flowing mortar. Finally, the mixture was cast in the mold and compacted with a mechanical vibration table until there was no bubble and covered with a layer of film. After 48 h in a room, the samples were removed from the molds and subjected to four different curing conditions.

### 2.3. Test Methods

#### 2.3.1. Mechanical Tests

**Figure 3:**
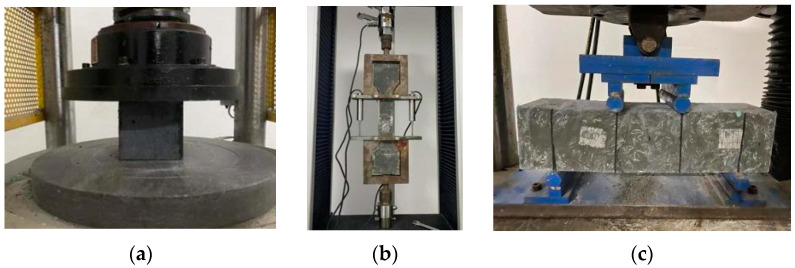
Mechanical property loading test of manufactured sand UHPC. (**a**) Compressive test. (**b**) Uniaxial tensile test. (**c**) Bending test.

The mechanical properties of the specimen are characterized according to the compressive, tensile and bending strength of specification T/CECS 864-2021 [38]. The compressive strength was performed at a loading rate of 1.2 MPa/s on 100 mm × 100 mm × 100 mm specimens. The samples were tested by YAW-3000 (microcomputer controlled electrohydraulic pressure testing machine) at 3, 7, 14, 28 and 56 days for four different curing conditions; the loading process is shown in Figure 3a. The uniaxial tensile strength was performed at a loading rate of 0.2 mm/min on dog-bone specimens. The samples were tested by WDW-100 (microcomputer controlled electronic universal testing machine) at 28 days for four different curing conditions; Lh-S05 tension sensor, YWC-100 strain displacement sensor and DN3816N static stress-strain system are used to collect and record the information of load and displacement, respectively; the loading process is shown in Figure 3b. The bending strength was performed at a loading rate of 0.1 mm/min on 100 mm × 100 mm × 400 mm specimens. The samples were loaded by four-point bending and tested by WAW-600B (microcomputer controlled electrohydraulic pressure testing machine) at 28 days for four different curing conditions; the loading process is shown in Figure 3c.

#### 2.3.2. Steel Fiber Pullout Test

**Figure 4:**
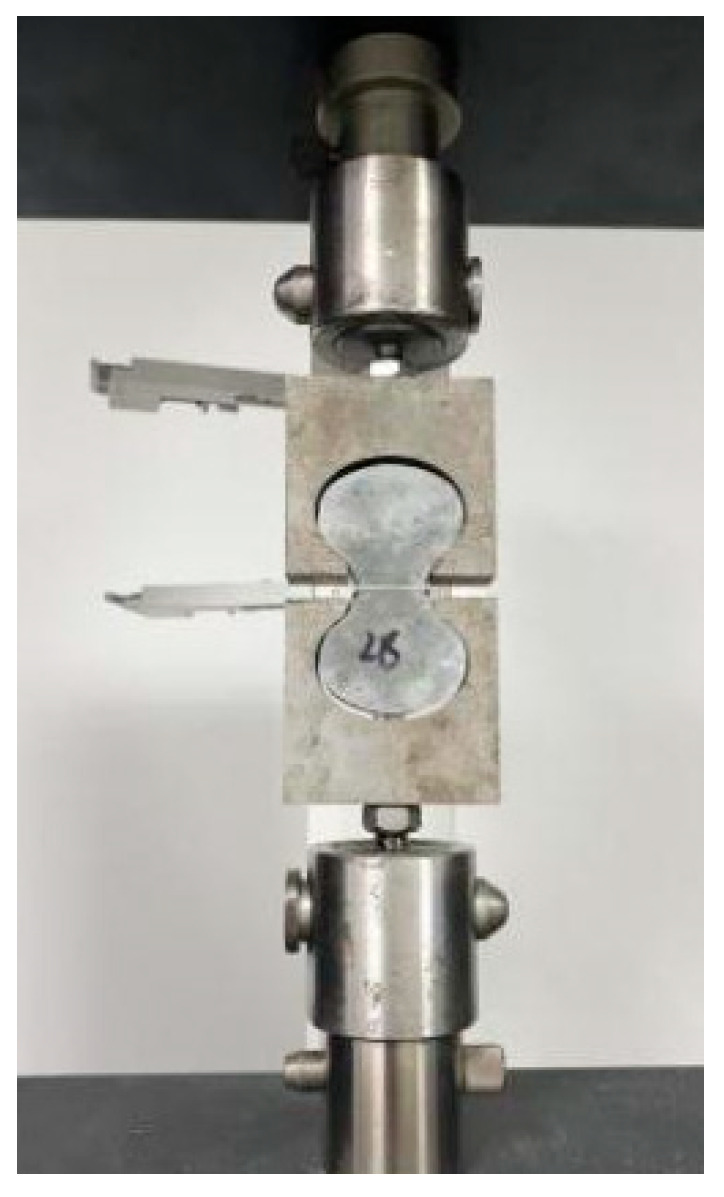
Steel fiber pullout test.

A steel fiber pullout test was used to study 3, 7, 14 and 28 days bond strength between steel fiber and matrix according to specification CECS 13-2009 [39]. The 8-shaped specimens were used for the pullout test, which was placed a 0.5–1 mm thick plastic diaphragm at the minimum section with four round holes. Four steel fibers were placed in the round holes and fixed with a spacing of 15 mm. The steel fiber on both sides of the diaphragm was at the embedded end and the fixed end, respectively; the length of the embedded end was 5 mm, and five specimens were tested in each group. The test was performed at a loading rate of 0.5 mm/min by WDW-20 (microcomputer controlled electronic universal tensile testing machine), and stopped when steel fiber slip exceeded 2.5 mm, the loading process is shown in Figure 4.

The bond strength between steel fibers and the UHPC cement matrix, *f*_fb_, can be calculated according to Equation (1). The pullout work, *W*_con_, which can be reflected by the area enclosed by the Load-slip curves and x axes, can be calculated according to Equation (2).

(1) $$f_{\mathrm{fb}}=\frac{F_{\max}}{4u_{f}l_{\mathrm{em}}}$$

(2) Wcon=∫Fdx

In Equation (1), *F*_max_ is the maximum pullout load, $\mu_{f}$ is fiber diameter and *l*_em_ represents the embedment length of the target fiber in the concrete matrix.

#### 2.3.3. Microscopic Test

The samples used for the microscopic test were taken from UHPC specimens with four different curing methods and 28 days age. Scanning electron microscopy (SEM, Hitachi S4800, Tokyo, Japan) was used to analyze the morphology and the internal microstructure of concrete under different curing conditions. Small pieces of samples (about 3 mm thick at least) were selected, and the surface of the samples was sprayed with Au to make them better conductive. The test condition was in a vacuum and the test voltage was 15 kV. X-ray diffraction (XRD, Rigaku Ultima IV, Tokyo, Japan) using a diffractometer at 40 kV and 35 mA. UHPC samples were ground to 200 mesh in a mortar before the test. The pore structure of the UHPC samples was analyzed using mercury intrusion porosimetry (MIP, Micromeritics AutoPore IV-9500, Atlanta, GA, USA). Firstly, the specimens were broken into approximately 5 mm pieces, and then to stop hydration the specimens were soaked in ethyl alcohol. The samples were dried at 50 °C in a drying oven for 20 h before experiment. The specimen tests were then carried out under high pressure of 413.70 MPa and low pressure of 0.28 MPa, respectively.

## 3. Results and Discussion

### 3.1. Mechanical Properties

#### 3.1.1. Compressive Strength

**Figure 5:**
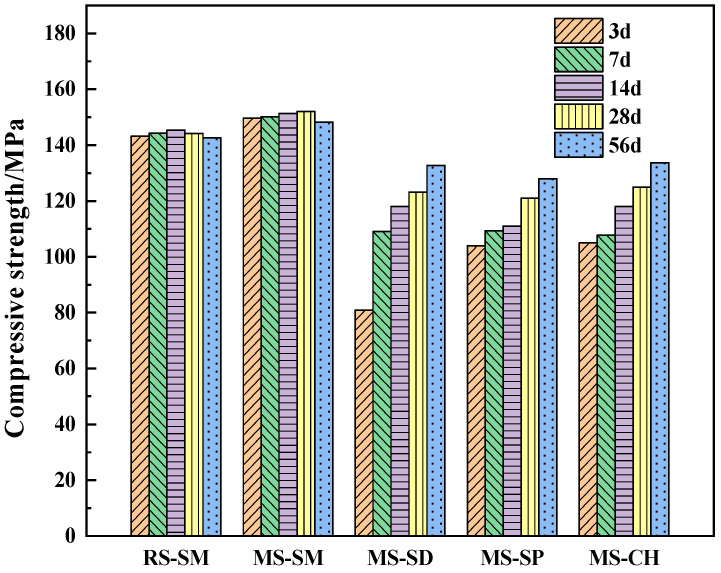
Compressive strength of manufactured sand UHPC at different ages under different curing methods.

The results of the UHPC compressive strength test are shown in Figure 5. As can be seen, under SM, the compressive strength of the MSUHPC developed rapidly, and the early compressive strength was much higher than that under the other three curing methods [40]. At the curing age of 3 days, the compressive strength of the MSUHPC under SM reached 149.7 MPa, while the compressive strength under SD, SP and CH were 80.9 MPa, 107.0 MPa and 105.0 MPa, respectively. Under SM, the later compressive strength of UHPC increased less, and the compressive strength even decreased slightly at the age of 56 days. This is because early hydration rate of UHPC is faster under the SM, and the hydration products quickly wrap the cement particles, which leads to the limited development of the later strength. Compared with normal temperature curing, SM promotes the formation of early hydration product C-S-H gel, which has longer molecular chain. However, when the molecular chain of C-S-H gel is too long, it will hinder the continuous formation of later hydration products and affect the later strength development of MSUHPC [41,42].

With the increase of age, the gap between the compressive strength of MSUHPC with other three curing methods and SM is gradually narrowed. CH can better improve the compressive strength of MSUHPC. Among them, the size rule of 28 days compressive strength of the material is as follows: CH > SD > SP, reaching 82.2%, 81.1% and 79.6% of the compressive strength of SM at the same age, respectively. When the curing age was 56 days, the compressive strength of CH, SD and SP reached 90.2%, 89.6% and 86.3% of that of SM, respectively.

Under the SM method, the compressive strength of MSUHPC at all ages is higher than that of RSUHPC. On the one hand, because of the high strength of the MS itself, it has the characteristics of rough surface, irregular shape and many corners, and has strong mechanical bite force between the cementitious material, so the compressive strength is higher than that of RSUHPC [43]. On the other hand, because the MS contains stone powder and has the nucleation effect, it can react with C_3_S and C_2_S in cement clinker to produce calcium carboaluminate hydrate, which makes the structure of UHPC more compact [44].

#### 3.1.2. Bending Strength

**Figure 6:**
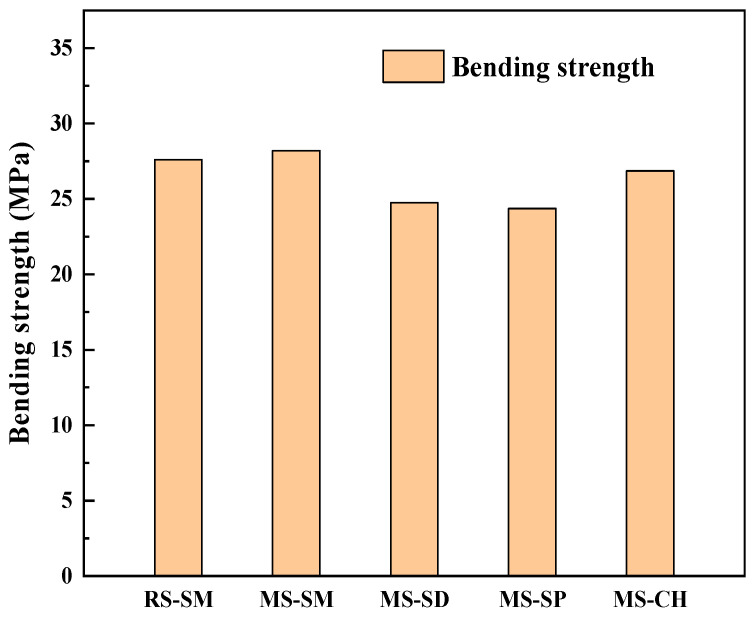
Bending strength of manufactured sand UHPC under different curing methods.

The results of the UHPC bending strength test are shown in Figure 6. As can be seen, the development law of bending strength and compressive strength of UHPC is similar. The bending strength of UHPC under SM was the highest. Compared with SM, the bending strength of UHPC under CH, SD and SP decreased by 4.8%, 12.2% and 13.6%, respectively. Under the SM, the bending strength of the MSUHPC is 2.17% higher than that of the RSUHPC.

#### 3.1.3. Uniaxial Tensile Property

**Figure 7:**
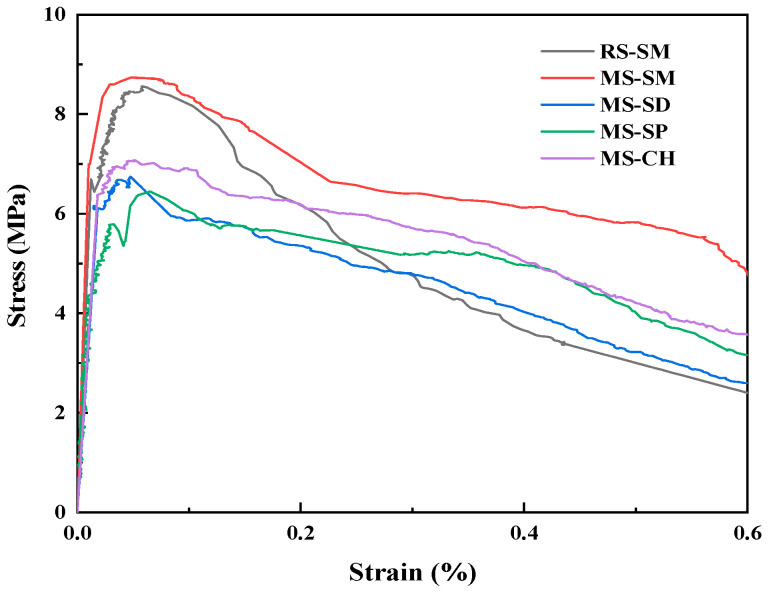
Tensile stress-strain curve of manufactured sand UHPC under different curing methods.

The tensile stress-strain curves of MSUHPC under different curing methods are shown in Figure 7.

The main performance indexes of the material in uniaxial tensile test include initial cracking strength (stress corresponding to the initial cracking of the matrix), tensile strength (ultimate tensile stress) and the ratio of tensile strength to initial cracking strength, as shown in Table 6.

Combined with Figure 7 and Table 6, it can be seen that there is no significant difference in the tensile properties of MSUHPC under SD, SP and CH; the initial cracking strength is in the range of 6.10~6.23 MPa, the tensile strength is in the range of 6.60~7.03 MPa, and the difference is within 7%. Under SM, the MSUHPC has higher initial cracking strength and tensile strength, which are increased by more than 19% compared with the other three curing methods. This is due to the promoting effect of SM on the hydration reaction, and more hydration products are generated at the fiber—matrix interface, which increases the bonding force between the two, thereby improving the initial crack strength and tensile strength of the matrix. Compared with SD and SP, the MSUHPC has higher tensile strength under CH provides sufficient moisture for the MSUHPC and Ca(OH)_2_ required for pozzolanic reaction, so that the hydration of the MSUHPC more sufficient and the matrix is more compact. Replacing RS with MS can slightly enhance the crack resistance of UHPC. Under SM, the initial cracking strength and tensile strength of RSUHPC are 7.44 MPa and 8.54 MPa, respectively, which are 4.0% and 2.1% lower than those of MSUHPC. In addition, the ratio of tensile strength to initial cracking strength of MSUHPC under the four curing methods is greater than 1.1, according to the classification of tensile properties in T/CECS 10107-2020, MSUHPC strain hardening characteristics under various curing methods, which can well explain the strain hardening phenomenon in uniaxial tensile process.

### 3.2. Bond Strength of Steel Fiber—UHPC Matrix

**Figure 8:**
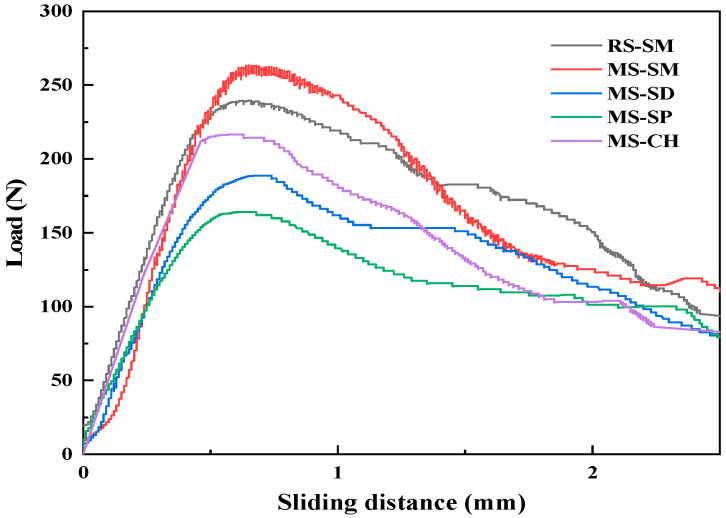
Load-slip curve of steel fiber and UHPC matrix.

**Figure 9:**
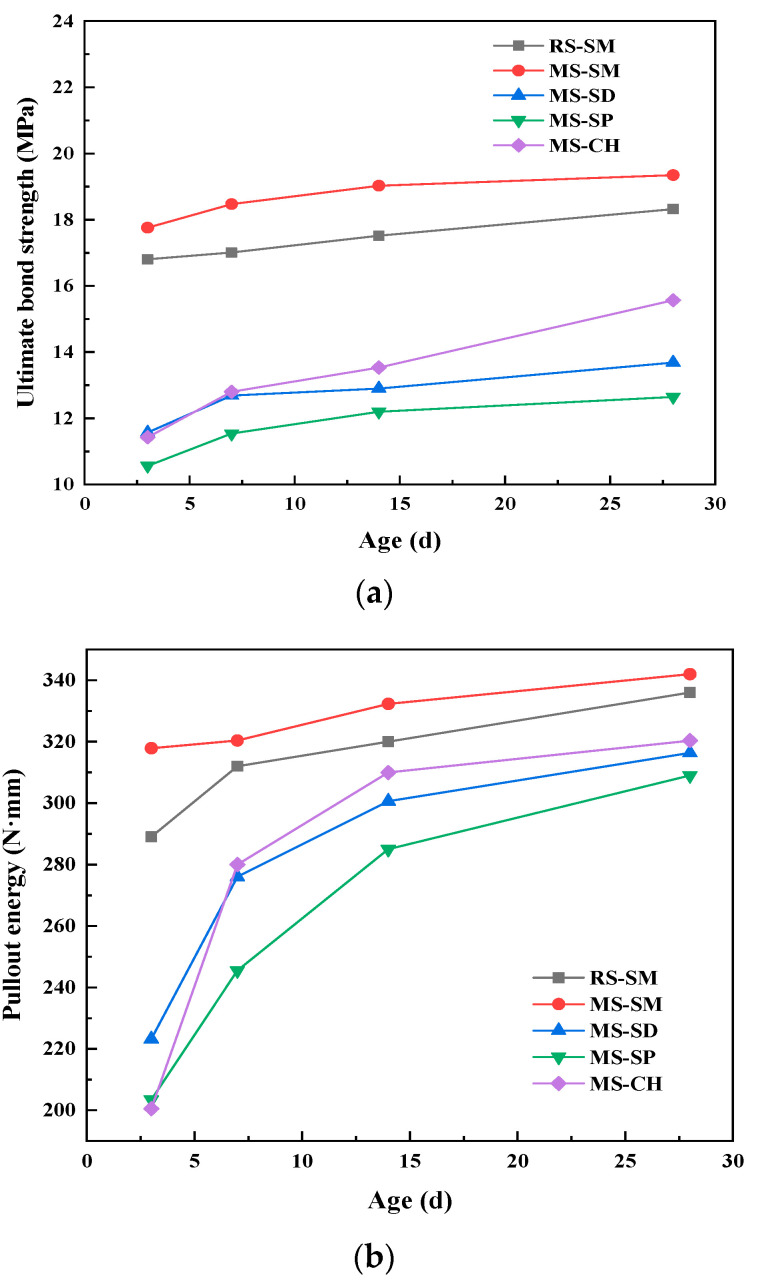
Bonding properties of manufactured sand UHPC at different ages under different curing methods. (**a**) Ultimate bond strength. (**b**) Pullout energy.

The load–slip curves of 28 days steel fiber and UHPC matrix under different curing methods are shown in Figure 8. Under different curing methods, the ultimate bond strength—pullout energy relationship curve between steel fiber and UHPC matrix at each age is shown in Figure 9.

The bonding properties and force failure characteristics of steel fibers and UHPC matrix are mainly related to three factors: steel fiber, UHPC matrix and the bonding force between them [45]. The curing method can change the strength and shrinkage characteristics of the UHPC matrix itself and then affect its bonding force to the steel fiber. It can be seen from Figure 8 and Figure 9 that under different curing methods, when the curing age increases from 3 days to 28 days, the load-slip curves of steel fiber and matrix are basically consistent. The ultimate load of the load-slip curve of steel fiber and MSUHPC matrix and the size of the area enclosed by the curve and the abscissa are: SM > CH > SD > SP; this shows that under SM, the fiber—matrix pullout energy is the highest, and the bonding performance of steel fiber and MSUHPC is the strongest. This also shows that the fiber—matrix pullout energy increases with the strength of the cemented matrix [46]. Under SM, compared with RSUHPC, steel fiber and MSUHPC matrix have higher ultimate load and better fiber—matrix bonding performance.

Under the four curing methods, the ultimate bond strength and fiber pullout energy of steel fiber and MSUHPC increased with the increase of curing age. Under SM, when the curing age is 3 days, the bond strength and fiber pullout energy of steel fiber and MSUHPC matrix are significantly higher than those of the other three curing methods, and the later strength and energy growth level are relatively flat. This is because SM can promote the hydration reaction at an early stage, thereby increasing the strength of the cementitious matrix. Under SD, SP and CH, with the increase of age, the hydration degree of the MSUHPC matrix is also increasing, and its ultimate bond strength with steel fiber and fiber pullout energy have a high increase. Under SM, the ultimate bond strength and fiber pullout energy of steel fiber and MSUHPC matrix increased slightly, and the 28 days age increased by 8.9% and 7.68% compared with the 3 days age, respectively. However, the ultimate bond strength of the other three curing methods increased by more that 16%, and the fiber pullout energy increased by at least 41%. From 3 days to 28 days, the ultimate bond strength and pullout energy increased the most under CH mode, by 36.2% and 57.8%, respectively.

### 3.3. Mechanism Analysis

#### 3.3.1. SEM Experiment

**Figure 10:**
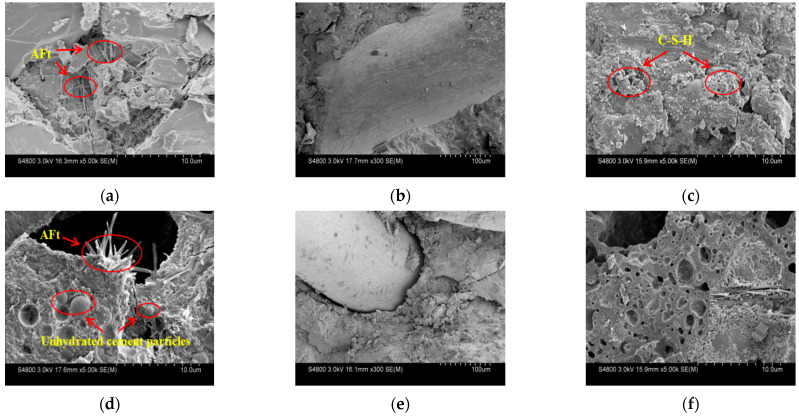
SEM images of manufactured sand UHPC samples under different curing methods. (**a**) RS-SM; (**b**) MS-SM; (**c**) MS-CH; (**d**) MS-SD; (**e**) MS-SP; (**f**) MS-SP.

The microscopic morphologies of MSUHPC under different curing methods are shown in Figure 10. Under SM, a large amount of acicular substance can be observed in RSUHPC, which is the acicular ettringite (AFt) formed by the further reaction of calcium aluminate hydrate (C_3_AH_6_) produced in the cement hydration process with gypsum in the cement (Figure 10a). The MSUHPC hydration products are tightly wrapped with steel fibers and have compact microstruture (Figure 10b). Therefore, MSUHPC exhibits better mechanical properties and stronger fiber—matrix adhesion than RSUHPC. Under CH and SD, a large amount of C-S-H gel and acicular ettringite (AFt) were generated in the MSUHPC, and many incompletely hydrated particles were also observed (Figure 10c,d), indicating that the hydration reaction of the cementitious material is insufficient. Under SP, the MSUHPC has many pores, relatively loose structure and relatively weak bonding force with fiber. Ca(OH)_2_ crystal and more spherical fly ash particles can be obviously observed in the matrix, indicating that the degree of secondary hydration reaction is low (Figure 10e,f).

#### 3.3.2. XRD Experiment

**Figure 11:**
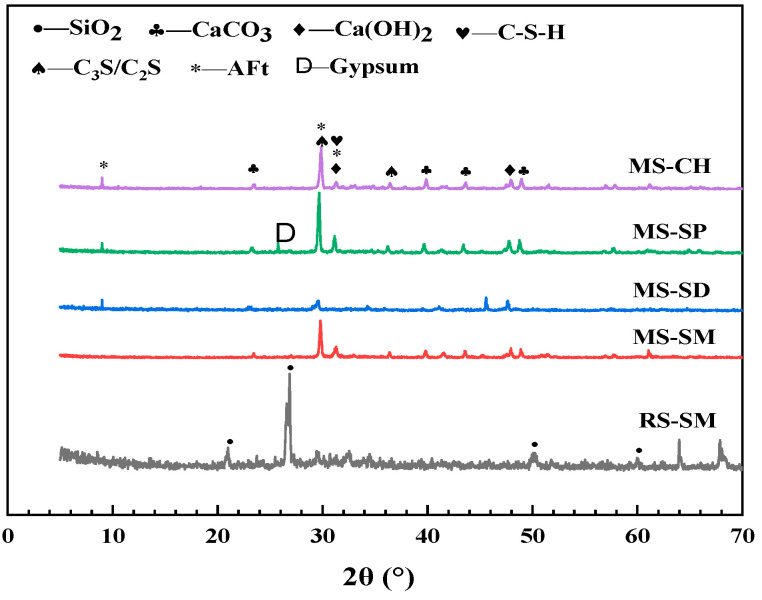
XRD diffraction patterns of manufactured sand UHPC under different curing methods.

The XRD patterns of MSUHPC under different curing methods are shown in Figure 11. The crystal phases measured mainly include SiO_2_, CaCO_3_, Ca(OH)_2_, C_3_S, C_2_S, AFt and C-S-H. Among them, the MSUHPC uses limestone MS as the fine aggregate, so a strong CaCO_3_ diffraction peak is detected, while the RSUHPC uses the RS as the fine aggregate, so a strong SiO_2_ diffraction peak is detected. The Ca(OH)_2_ diffraction peak under SM is relatively weak, which confirms the promotion effect of SM on secondary hydration reaction of UHPC matrix. C_3_S and C_2_S diffraction peaks were found in each group of MSUHPC, because the water–binder ratio of MSUHPC was extremely low, the cement hydration reaction was insufficient, and there was unhydrated cement clinker in the sample. Among them, the diffraction peaks of C_3_S and C_2_S under SP are relatively strong, indicating that due to the relatively low hydration degree of cementitious materials under SP, there are more unhydrated cement clinkers.

#### 3.3.3. MIP Experiment

**Figure 12:**
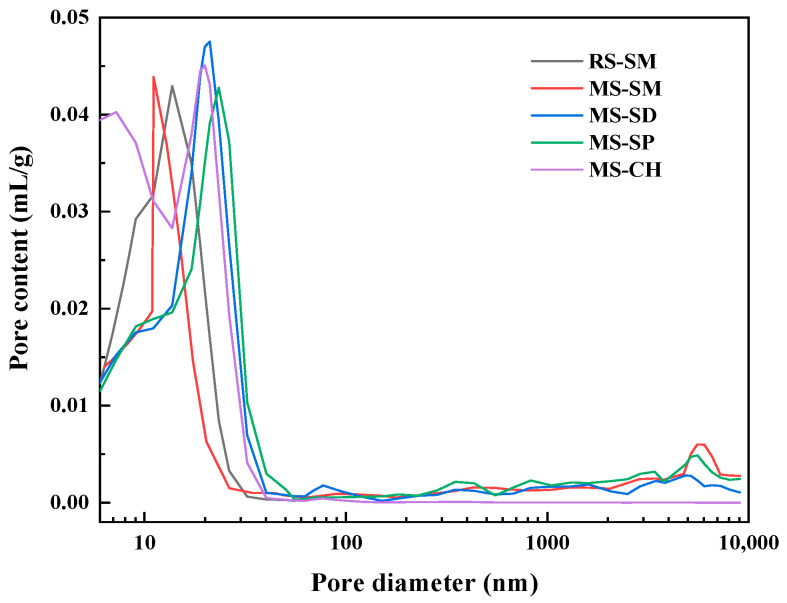
Pore size distribution of manufactured sand UHPC under different curing methods.

The pore size distribution curves of MSUHPC under different curing methods are shown in Figure 12. The porosity, the most probable pore size and the pore fractal dimension are shown in Table 7.

It can be seen from Table 7 that the total porosity of MSUHPC under different curing methods is as follows: SM < CH < SD < SP. During SM, high temperature accelerates cement hydration and promotes pozzolanic reaction to generate more hydration products. The material structure is more compact, the total porosity is the lowest, and the most probable pore size is the smallest. The porosity index under CH and SD is relatively close, and the porosity is 2.74 times and 2.81 times that under SM, respectively. The porosity of SP is the highest, and the porosity is 3.06 times that of SM. This is because SP cannot continuously provide water for the hydration process of MSUHPC, resulting in the slowing down of its hydration rate and the reduction of hydration products.

The fractal dimension can reflect the pore characteristics of concrete and quantitatively describe the roughness and complexity of concrete pore surface [47,48]. The larger the pore fractal dimension, the more complex the spatial geometric features representing the pores [49]. Comparing the pore fractal dimension of MSUHPC under different curing methods, it is found that SM is the largest, followed by CH and SD, and SP is the smallest. This shows that more hydration products are produced during SM, which makes the matrix structure more compact, the macropores decrease, the small pores increase and the specific surface area of the pores increases. The fractal dimension of CH and SD is close. The fractal dimension of SP is the lowest, and there are relatively more macropores in the material, and the pore complexity is low. Under SM, the pore fractal dimension of the MSUHPC is larger than that of the RSUHPC, which indicates that the pore distribution inside the MSUHPC material is more complex, and the proportion of small pores is higher; this is because the RS has a more regular shape and a smoother surface than the MS, and produces less pore complexity when combined with the matrix.

## 4. Conclusions

In this research, UHPC was prepared by fully replacing RS with MS. The effects of different curing methods on the mechanical properties of MSUHPC and the bonding properties between steel fibers and MS were analyzed through mechanical property tests combined with microscopic tests. The following conclusions were drawn:

(1) Under SM, the compressive strength of MSUHPC increased greatly in the early stage, and the later strength increased less. The 28 days compressive strength increased by only 1.5% compared with the 3 days compressive strength. The 28 days compressive strength of CH, SD and SP increased by 19.1%, 52.3% and 16.3% respectively compared with 3 days compressive strength. In addition, the compressive strength of four curing methods was only negatively increased in SM at 56 days. The development law of bending strength and compressive strength of MSUHPC under different curing methods is similar, but due to the significant influence of steel fiber on the bending properties, the difference in bending strength of the four curing methods is small.
(2) Under SD, SP and CH methods, the tensile properties of MSUHPC do not show significant differences. Compared with the other three curing methods, SM method increased the initial crack strength and tensile strength of MSUHPC by more than 19%. The ratio of tensile strength to initial crack strength of MSUHPC is greater than 1.1 under all four curing methods and shows strain hardening characteristics. The tensile properties of MSUHPC are slightly better than RSUHPC under SM method.
(3) The ultimate bond strength of steel fiber and MSUHPC matrix under SM has a small increase in the later period, and the 28 days ultimate bond strength only increases by 8.9% compared with the 3 days ultimate bond strength. The 28 days ultimate bond strength of CH, SD and SP increased by 36.2%, 18.3% and 16.5% respectively compared with 3 days ultimate bond strength. Moreover, compared with the other three curing methods, SM shows that the increase of fiber pullout energy of MSUHPC is more different. The SM 28 days fiber pullout energy increases by 7.7% compared with 3 days fiber pullout energy, while the other three curing methods all increase by 40%.
(4) Under SM, the porosity and the most propable pore diameter of MSUHPC were lower, and the Ca(OH)_2_ diffraction peak is relatively weak, indicating that SM promoted the secondary hydration reaction of UHPC matrix. However, SM has an adverse effect on the long-term performance of concrete, so it needs to be kept wet in the later stage to eliminate its adverse effects. Under SD, CH and SP, the porosity and the most probable pore diameter of MSUHPC are relatively close, but larger than the SM method. The pore fractal dimension of MSUHPC is the largest under SM, indicating that the pore distribution in the material is more complex and the proportion of small pores is higher.

## Figures and Tables

**Table 1:** Chemical and physical properties of cement.

Properties		Value	Range ^1^
Physical properties	Specific surface area (m^2^/kg)	386	≥300
	Initial set (min)	170	≥45
	Final set (min)	222	≤600
Compressive strength	28 days (MPa)	55.6	≥52.5
Flexural strength	28 days (MPa)	7.5	≥7.0
Chemical properties	Stability	Qualified	-
	Loss on ignition (%)	2.30	≤5.0
	SO_3_ (%)	2.33	≤3.5
	Cl^−^ (%)	0.036	≤0.06
	Mixed admixture (%)	6.0	>5 and ≤20

^1^ GB 175-2007 [36].

**Table 2:** Physical properties of steel fiber.

Index	Length (mm)	Diameter (mm)	Density (g/cm^3^)	Tensile Strength (MPa)
Unit value	13	0.2	7.8	≥2850

**Table 3:** Physical properties of natural river sand.

Properties		Value
Physical properties	Fineness modulus	2.10
	Bulk density (kg/m^3^)	1570
	Tight density (kg/m^3^)	1740
	Apparent density (kg/m^3^)	2550
	Water absorption of the dry saturation surface (%)	1.3
	Dust content (%)	0.5

**Table 4:** Physical properties of manufactured sand.

Properties		Value
Physical properties	Fineness modulus	2.10
	Bulk density (kg/m^3^)	1720
	Tight density (kg/m^3^)	1900
	Apparent density (kg/m^3^)	2560
	Water absorption of the dry saturation surface (%)	1.0
	Crash value index (%)	5.4
	Crusher dust content (%)	4.5
	Methylene blue value (g/kg)	0.5
	Soundness (%)	6

**Table 5:** Mixture proportions of UHPC (kg/m^3^).

Group	Cement	Fly Ash	Silica Fume	Superplasticizer	MS	RS	Steel Fiber	Water	Slump Spread (mm)
MS	1000	100	200	40.2	1200	-	235.5	221	425
RS	1000	100	200	40.2	-	1200	235.5	221	395

**Table 6:** Tensile performance index.

Specimen NO.	*F*_te_ (MPa)	*F*_cu_ (MPa)	*F*_te_/*F*_cu_
RS-SM	7.44	8.54	1.15
MS-SM	7.75	8.72	1.11
MS-SD	6.15	6.72	1.10
MS-SP	6.10	6.60	1.10
MS-CH	6.23	7.03	1.14

Notes: *F*_te_ represents the initial crack strength; *F*_cu_ represents the tensile strength.

**Table 7:** Pore index of manufactured sand under different curing methods.

Specimen NO.	Porosity (%)	Most Probable Pore Size (nm)	Fractal Dimension
RS-SM	2.97	13.74	2.855
MS-SM	2.48	11.10	2.872
MS-SD	6.99	21.09	2.821
MS-SP	7.59	23.43	2.698
MS-CH	6.79	19.93	2.827

## Data Availability

The general data are included in the article. Additional data are available on request.
